# Sepsis-induced hypocholesterolemia is linked to low cardiomyocyte membrane cholesterol and impaired catecholamine responsiveness

**DOI:** 10.1186/s13054-025-05638-7

**Published:** 2025-09-25

**Authors:** Anna Kleyman, Walter Pisciotta, Charlotte Gaupp, Waqas Khaliq, Daniel Hofmaenner, David Brealey, Bernardo Bollen Pinto, Davide Tommaso Andreis, Mark Gerard Waugh, Miranda J. Melis, Muska Miller, Klea Mehmetaj, Michael Bauer, Adrian Press, Mervyn Singer

**Affiliations:** 1 Bloomsbury Institute of Intensive Care Medicine, Division of Medicine, University College London, Cruciform Building, Gower St, London, WC1E 6BT UK; 2 Department of Anaesthesia and Intensive Care, IRCCS-ISMETT (Istituto Mediterraneo per Trapianti e Terapie ad alta specializzazione), Palermo, Italy; 3 Department of Internal Medicine, Spital Limmattal, Schlieren, CH-8952 Switzerland; 4 Institute of Intensive Care Medicine, University Hospital Zurich, Zurich, Switzerland; 5 Dept of Critical Care, University College London Hospitals NHS Foundation Trust, London, UK; 6 Division of Anaesthesiology, Geneva University Hospitals, Geneva, Switzerland; 7 S.C. Anaesthesia and Intensive Care, Legnano Hospital, ASST Ovest Milanese, Milan, Italy; 8 Division of Medicine, University College London, London, UK; 9 Dept of Anesthesiology and Intensive Care Medicine, Jena University Hospital, Jena, Germany

**Keywords:** Cholesterol, Sepsis, Cardiomyopathy, Myocardial depression, Catecholamine

## Abstract

**Background:**

Sepsis-induced cardiomyopathy (SIM) is characterized by myocardial dysfunction, diminished catecholamine responsiveness and worse outcomes. Hypocholesterolemia is also a well-recognized prognosticator of poor outcomes in sepsis. In vitro physiology/pharmacology studies indicate that low cholesterol levels within the cardiomyocyte membrane regulate ß-adrenergic receptor activity. We therefore hypothesized that cardiomyocyte membrane cholesterol levels are reduced in sepsis and this contributes to SIM.

**Methods:**

Cardiovascular biomarkers and plasma lipid profiles measured sequentially (6, 24 and 72 h) in a fluid-resuscitated rat model of fecal peritonitis were compared against those measured in 27 septic patients on Days 1–3 of ICU admission. In separate studies, rat hearts were excised at the same time points for measurement of cardiomyocyte membrane cholesterol and downstream adrenergic signaling. In a final study, the impact of a 15-hour infusion of cholesterol, either given as HDL-cholesterol or liposomal cholesterol, commencing at 6 h post-sepsis induction, on dobutamine responsiveness and cardiomyocyte membrane cholesterol levels was assessed.

**Results:**

The magnitude of fall in stroke volume, rise in heart rate, plasma troponin and BNP, and fall in plasma HDL-cholesterol on ICU Day 1 in septic patients and at 6 h in the rat model all prognosticated for poor outcomes. In parallel, cardiomyocyte membrane cholesterol fell in the rats, more so in poor prognosis animals, with a blunted inotropic response to dobutamine, indicative of SIM. Cholesterol administration restored cardiomyocyte membrane cholesterol, dobutamine responsiveness and adrenergic signaling.

**Conclusions:**

In a long-term rat model of sepsis, that parallels changes seen in septic patients, cardiomyocyte membrane cholesterol fell with associated decreases in catecholamine responsiveness. These features could be restored by cholesterol infusion, suggesting potential utility as a therapeutic.

**Supplementary Information:**

The online version contains supplementary material available at 10.1186/s13054-025-05638-7.

## Introduction

Sepsis-induced cardiomyopathy (SIM) is a well-recognized complication of sepsis and septic shock [1]. Features include systolic and/or diastolic dysfunction affecting left and/or right ventricles, reduced cardiac output, raised biomarkers of ventricular dysfunction and injury such as plasma B-type natriuretic peptide and troponins, and catecholamine hyporesponsiveness [2–8]. The pathogenesis of SIM is complex and multi-factorial [1]. Mechanisms suggested to explain catecholamine hyporesponsiveness include decreased expression of adrenergic receptors, desensitization of adrenergic receptors by activation of G-protein receptor kinase (GRK2), dysregulation of enzymes regulating downstream signaling such as phosphodiesterase PDE4 and protein phosphatase 2 A, and alterations in the effector apparatus, e.g. impaired calcium (Ca^2+^) trafficking and myofilament sensitivity [6–8].

We hypothesized that an important but previously unexplored mechanism underlying SIM is reduced cardiomyocyte cholesterol. Low plasma cholesterol levels have been long recognized to occur early in sepsis and other critical illnesses, with the degree of fall associated with worse outcomes [9]. Cholesterol has many functions within the body [10]; it is a precursor of steroid hormones, vitamin D and bile acids, it possesses antioxidant [11, 12] and anti-inflammatory properties [13] and is an integral component of the plasma membrane of all eukaryotic cells.

The plasmalemmal cholesterol concentration controls membrane structure and its biophysical properties, including fluidity and permeability. The plasmalemmal cholesterol concentration also regulates, both directly and indirectly, the localization and activity of numerous transmembrane and membrane-associated proteins, including transporters, ion channels and receptors [14–19]. Membrane cholesterol is mainly located within lipid rafts to which G protein-coupled receptors, such as the b-adrenergic receptor, are co-located [20–22]. Cholesterol plays a critical role in the organization and regulation of signaling from the receptors located in lipid rafts [23, 24]. ß-adrenergic receptors (ßAR) have cholesterol-binding sites [25, 26] and signal through lipid rafts [27–29]. Experimental depletion of cardiomyocyte membrane cholesterol results in dysregulation of adrenergic signaling [30, 31].

Our three-day fluid-resuscitated rat model of fecal peritonitis has many of the characteristics of human sepsis, including myocardial depression and decreased responsiveness to fluid and the b-adrenergic catecholamine, dobutamine [32–34]. Critically, prognostication can be accurately made as early as 6 h with lower stroke volume and higher heart rates measured in eventual non-survivors [32–34]. Using this model, we examined whether cardiomyocyte cholesterol depletion occurs during sepsis, whether this depletion correlates with illness severity and reduced dobutamine responsiveness, and whether restoring membrane cholesterol levels could reverse these effects. We assessed two cholesterol formulations, one bound to HDL and the other incorporated within liposomes. The underlying rationale was to determine whether the use of HDL offered additional effects over and above cholesterol as it possesses toxin-binding, anti-inflammatory and endothelium-protective effects [35–41]. To further validate the clinical relevance of our findings, we compared plasma lipid and cardiac biomarker levels from our animal model with those obtained from septic patients in intensive care.

## Materials and methods

Further detail of the model and methodologies used can be found in the Supplementary Materials.

### Patient study

The study was approved by the UK National Ethics Research Service Committee (reference 11/EE/0180). Patients admitted to the intensive care unit of University College Hospital, London, UK, with abdominal or chest sepsis, new-onset multi-organ dysfunction with a rise in Sequential Organ Failure Assessment (SOFA) score > 3 points, and predicted to stay more than three days were enrolled. Next-of-kin approval was obtained for blood sampling, with retrospective patient consent obtained on regaining mental competency.

Twenty ml arterial blood samples were placed into appropriate tubes within 6 h of ICU admission (day 0), and then repeated on days 1, 2 and 3. Plasma was separated, aliquoted and snap-frozen in liquid nitrogen. Demographic and concurrent clinical data were also collected and presented in the Supplementary Materials. Stroke volume was measured by esophageal Doppler flow velocimetry (CardioQ, Deltex Medical, Chichester, UK).

### Rat fecal peritonitis model studies

Male Wistar rats (Charles River, Margate, UK) weighing 325–400 g were used throughout. All experiments were performed under a Home Office Project License (PPL 70/7029) and local University College London (UCL) Ethics Committee approval. Experiments were performed in accordance with relevant guidelines and regulations.

Rats were maintained in separate cages with a 12-hour light-dark cycle. Under brief isoflurane anesthesia and buprenorphine analgesia, rats underwent tunneled internal jugular venous and carotid arterial cannulation with 0.96 mm outer diameter PVC tubing (Biocorp Ltd, Huntingdale, NSW, Australia). Both lines were tunneled subcutaneously to the nape of the neck. secured in place with two 3 − 0 silk sutures and attached to a swivel tether system (InsTech Solomon, Plymouth Meeting, PA, USA) in their cage. This system enabled unimpaired movement of the animal around its cage after recovery from anesthesia, with *ad libitum* access to food and water, and prevented line removal and tangling. Continuous blood pressure monitoring and blood sampling were performed through the arterial line, and fluid resuscitation and drug administration through the venous line.

Sepsis was induced by an intraperitoneal injection (4 µl/g body weight) of human fecal slurry diluted in saline. Intravenous fluid resuscitation (50:50 mix of 5% glucose/Hartmann’s solution) was commenced at a rate of 10 ml/kg/h from 2 h after sepsis induction and reduced to 5 ml/kg/h at 48 h. Animals underwent regular inspection using an in-house clinical severity scoring system (Supplementary Table 1). Experience with this model found that animals passing a severity threshold died within 2–4 h, so animals were humanely sacrificed if they reached this point.

Depending on the study, animals were briefly or terminally anesthetized at 6, 24 and 72 h for echocardiographic heart rate measurement and stroke volume (Vivid 7 Dimension and Vivid 10 S 10 MHz sector transducer, GE Healthcare, Bedford, UK) in a non-blinded manner. Blood samples (1 ml or up to 6 ml at experiment termination) were taken into appropriate heparinized or non-heparinized tubes at the same timepoints. After centrifugation, plasma samples were aliquoted and frozen at −80 °C until subsequent analyses.

**Fig. 1:**
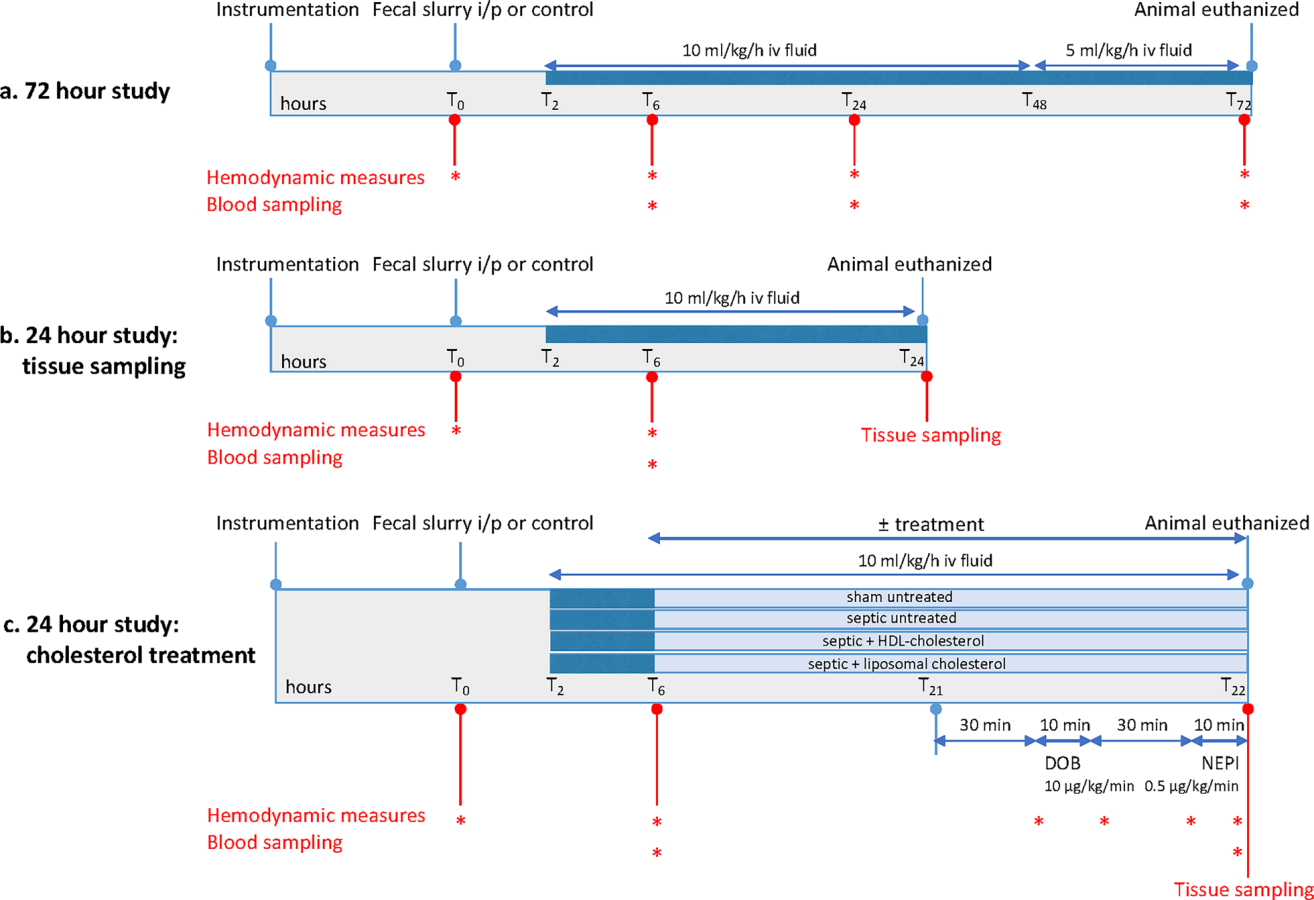
Design of three animal studies (**a**) 72 h study (**b**) 24 h tissue sampling study and **(c**) 24 h cholesterol treatment study i/p intraperitoneal; MAP mean arterial pressure; DOB dobutamine; NEPI norepinephrine

Three studies were performed, summarized below and depicted in Fig. 1:

## Study (a) confirmation of 72-hour outcome prognostication by echocardiography at 6 hours

Previous studies indicated that a low stroke volume (< 0.17 ml) measured at 6 h prognosticated 3-day mortality with positive and negative predictive values of 93% and 80%, respectively [32–34]. Mortality predominantly occurred between 16 and 40 h, with survivors showing clear signs of clinical recovery at 72 h with improved appearance, increased activity and interest in their environment, and increased food intake.

To confirm this prior finding, 16 animals (350 ± 25 g body weight) had sepsis induced as described above, with blood sampling and echocardiographic measurements performed at 6, 24 and 72 h. Six sham-operated animals were treated identically but did not receive a fecal slurry injection.

## Study (b) tissue sampling at 24 hours

Forty-eight male Wistar rats were subjected to the fecal peritonitis insult described above, with a further six animals acting as sham-operated controls. Stroke volume measured at 6 h was used to distinguish predicted survivors from non survivors. At 24 h, all surviving animals (6 sham, 19 predicted survivors, and 15 predicted non survivors) were terminally anesthetized, and then euthanized by exsanguination with collection of blood samples as above. Heart, liver and kidney were rapidly extracted, washed with ice-cold PBS, cut into small pieces, and then snap-frozen in liquid nitrogen.

## Study (c) impact of cholesterol infusions on cholesterol levels and catecholamine responsiveness

At 6 h post-sepsis induction and before echocardiographic evaluation, 41 septic rats were randomly allocated to untreated sepsis (*n* = 14), sepsis + HDL-cholesterol (HDL-C) (*n* = 12) or sepsis + liposomal cholesterol (*n* = 15) groups. Thirteen animals acted as sham-operated healthy controls. Within the background fluid infusion (10 ml/kg/hour = ~ 3.33 ml/hour), the cholesterol groups received over 15 h either bovine HDL-cholesterol (HDL-C) (600 µl bovine HDL containing 35 mg cholesterol, Lee Biosolutions, Maryland Heights, MI, USA) or liposomal cholesterol (1 ml liposomal solution containing 3.2 mg cholesterol, Jena University Hospital, Germany. For the details of preparation see Supplementary Materials). Solubility issues prevented higher concentrations of hydrophobic cholesterol from being administered via the current liposome formulation. At 21 h post-sepsis induction, animals were anesthetized with isoflurane. After a 30 min stabilization period, animals underwent baseline measures of blood pressure, stroke volume and heart rate. The ß-adrenergic agonist, dobutamine, was then infused at 10 µg/kg/min for 10 min, followed by repeat echocardiography and BP recording. After a 30 min washout period, this procedure was repeated but with a 10 min infusion of the alpha-adrenergic agonist, norepinephrine at 0.5 µg/kg/min. Animals were then euthanized by exsanguination with plasma, heart, liver and kidney samples collected and stored for subsequent analysis.

## Ex vivo measurements

### Human patient plasma measurements

Total, HDL and LDL/VLDL cholesterol, triglyceride (AU5800 analyzer, Beckman Coulter, High Wycombe, UK) and high-sensitivity troponin-T levels were measured by the Clinical Chemistry Department, Royal Free Hospital, London, UK, who also provided normal ranges for these assays. B-type natriuretic peptide (BNP) (E-EL-H0598, Elabscience) and the cytokines IL-6 and IL-10 (DY-506 and DY522, R&D Systems) were measured by ELISA in our laboratory following the manufacturers’ instructions.

### Rat plasma measurements

Total, HDL- and LDL-cholesterol and triglycerides in the 72 h study were also measured by the Royal Free Hospital Clinical Chemistry Department. In other studies, total cholesterol levels were measured by Amplex Red assay (Invitrogen A12216). BNP (RAB0386, Sigma-Aldrich), troponin T (E-EL-R0151, Elabscience), IL-6 and IL-10 (DY-506 and DY-522, R&D Systems) were measured by ELISA according to the manufacturers’ instructions.

### Cardiomyocyte, liver, and kidney membrane preparations and measurements

Frozen tissue was pulverized in liquid nitrogen, homogenized in Tris HCl Buffer pH 7.4 and sequentially centrifuged. The final membrane pellet was redissolved in the Triton X-100 buffer and used for measurement of protein concentration (Pierce™ BCA Protein Assay Kit, ThermoFisher Scientific 23225) and cholesterol (Amplex Red Cholesterol Assay kit, Invitrogen A12216). For the details, see the Supplementary Materials.

Separately, protein lysates were prepared from frozen heart tissue. The protein concentration was measured by BCA assay (ThermoFisher Scientific). Downstream markers of adrenergic signaling [30–32] – phosphotroponin I (pTnI) and phosphoERK2 (pERK2) (antibodies 4004 and 9101, Cell Signalling), and phosphofructokinase as a reference protein, were measured by Western blot (see Supplementary Materials for details). Results were evaluated by Image Studio Lite version 5.2.

### Statistical analyses

Prism (Version 9.5.1, GraphPad Software, Boston, MA, USA) was used for analysis. After normality testing, parametric (Student’s t-test) or non-parametric (Wilcoxon rank sum test) analyses compared matched or dependent samples. One- or two-way analysis of variance (ANOVA) was used where appropriate, with uncorrected *post-hoc* comparisons made using Tukey (parametric) or Kruskal-Wallis (non-parametric) tests if the overall ANOVA was positive. Pearson’s correlation coefficient was used to assess relationships. Sample sizes generally exceeded six per group as long experience with the rat model identifies this number is usually sufficient to show both clinically relevant and statistically significant results. On a few occasions, group sizes were below six due to a lack of sufficient samples or spoiled samples. Data are presented as mean ± standard deviation (parametric) or box and whisper plots (non-parametric).

## Results

### (i) prognostic biomarkers in the rat septic model mirrors that seen in human sepsis

#### Patients

**Fig. 2:**
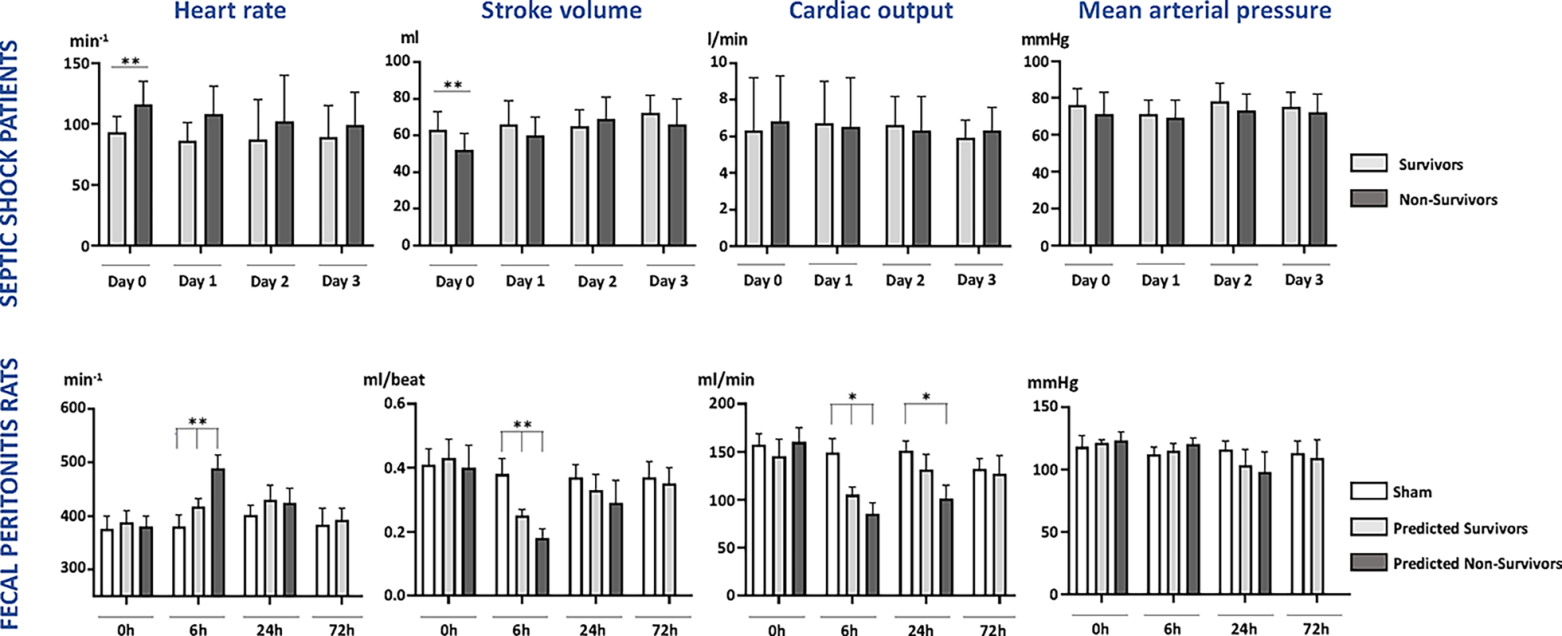
Hemodynamic data over three days in septic ICU patients (top row) and rats with fecal peritonitis (lower row). Hemodynamic data are shown as mean ± SD. Parametric analyses with post-hoc testing shown if overall ANOVA positive (*p < 0.05; **p < 0.01). Patient group sizes vary from 11 (survivor) and 12 (non-survivor) on Day 0, to 6 (survivor) and 9 (non-survivor) on Day 3 (numbers varied depending on use of cardiac output monitoring). Rat group sizes vary from 3–9 (six sham, seven survivors, nine non-survivors at 0 and 6 h and three at 24 h)

Twenty-seven patients admitted to intensive care with intra-abdominal sepsis or community-acquired pneumonia were enrolled, of whom 24 (89%) required vasopressor therapy and 12 (44%) subsequently died within 28 days. Demographic and clinical data are shown in Supplementary Table 1, with patient data separated into survivors and non-survivors. On Day 0, compared to eventual survivors, heart rate was significantly higher (*p* < 0.001) and stroke volume significantly lower (*p* = 0.008) in non-survivors (Fig. 2, top panel).

**Fig. 3:**
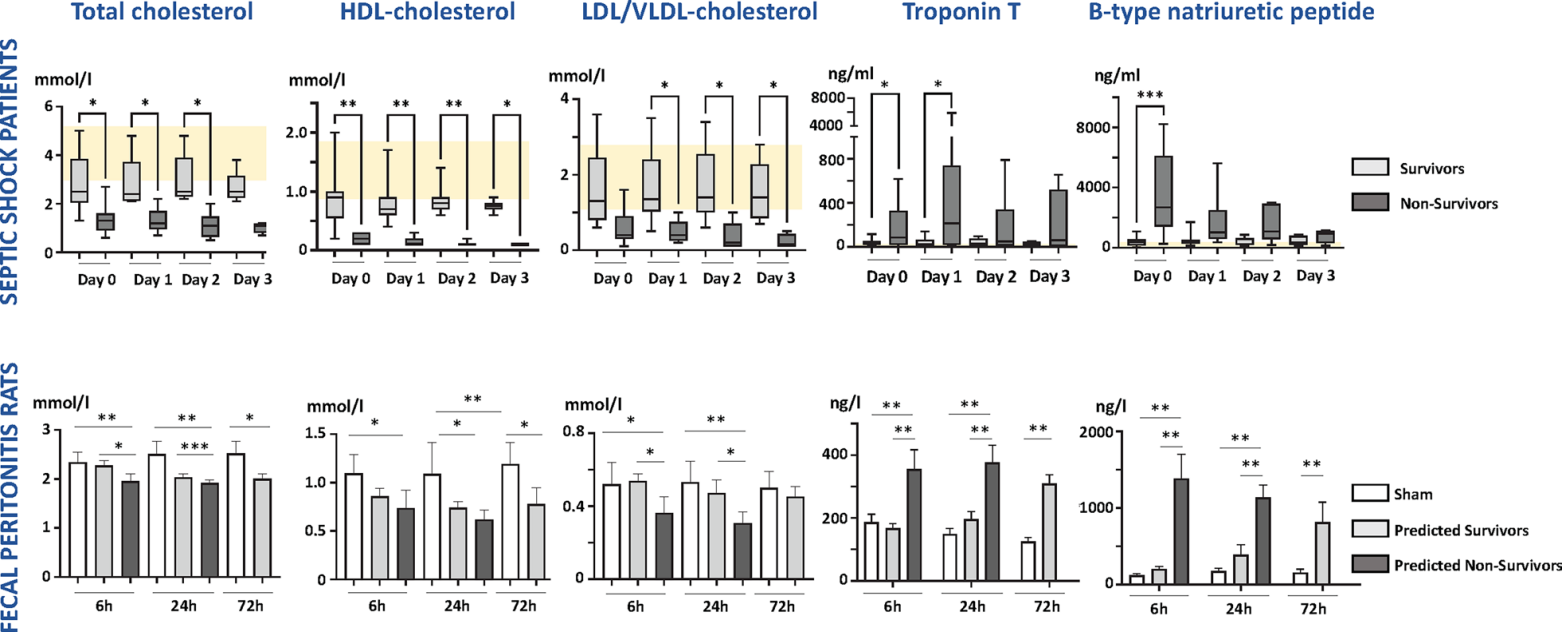
Total, HDL and LDL Cholesterol and markers of cardiac injury (troponin T) and ventricular dysfunction (B-type natriuretic peptide) over three days in septic ICU patients (top row) and rats with fecal peritonitis (lower row). Hemodynamic data shown as mean ± SD (parametric) or box and whisper plots (non-parametric). Repeated measures 2-way ANOVA with post-hoc testing shown if overall ANOVA positive (*p < 0.05; **p < 0.01, ***p < 0.001). Patient group sizes: 13, 12, 9 and 8 survivors on Days 0–4, respectively; 11, 9, 6, and 4 non-survivors on Days 0–4, respectively. Rat group sizes vary 5–8 rats/group. Yellow-shaded area denotes normal laboratory ranges in humans. HDL high-density lipoprotein; LDL/VLDL = low-density/very low-density lipoprotein

Figure 3 demonstrates low-to-subnormal levels of total, HDL and LDL/VLDL cholesterol from ICU admission, with no change over the next three days (ANOVA). Levels were markedly reduced in non-survivors compared to survivors (*p* < 0.01). Triglyceride levels were in the low to subnormal range throughout the first 3 days, with no difference between survivors and non survivors (Supplement Fig. 2).

Troponin-T and BNP levels were elevated above the normal range in most patients, particularly in non-survivors at early timepoints, albeit only statistically significant between survivors and non-survivors for BNP at Day 0 and troponin at Days 0 and 1 (Fig. 3). IL-6 and IL-10 levels were elevated throughout, compared to normal ranges (< 100 pg/ml for IL-6, < 50 pg/ml for IL-10), especially at early timepoints in eventual non-survivors. This was only statistically significant between survivors and non-survivors for IL-10 at Day 0 (Supplement Fig. 2). Total and HDL cholesterol levels inversely correlated with BNP but not with troponin over Days 0–3 (Supplement Fig. 3).

#### Rats

In the 72-hour rat fecal peritonitis model [Study (a), 6 (37.5%) of 16 animals died within 24 h, and 9 (56.3%) by 72 h. Mortality predominantly occurred between 18 and 36 h (Supplement Fig. 1). Animals surviving beyond 48 h showed clear signs of clinical recovery at 72 h with increased activity, a normal eating pattern and less piloerection. In the septic animals, falls in stroke volume and cardiac output and tachycardia were more prominent in septic non-survivors, and most apparent at 6 h (Fig. 2, lower panel). Values had normalized by 72 h in the surviving animals. For 72 h survival prognostication determined by 6-hour echocardiography, receiver operating characteristic curves identified a heart rate cut point of 460 beats/min with a sensitivity of 0.88 and specificity of 0.92, while a stroke volume < 0.19 ml showed a sensitivity of 0.90 and specificity of 0.86. Based on the above criteria, all six sham-operated control animals and all seven predicted survivor rats survived to 72 h.

Early falls occurred in plasma levels of total, HDL and LDL/VLDL cholesterol after sepsis initiation, especially in non-survivors in whom significance was achieved as early as 6 h (Fig. 3). Levels in survivors had not normalized by 72 h. Triglyceride levels were significantly lower in non-survivors compared to sham animals at 6 h, with no significant difference seen thereafter. Triglyceride levels were maintained throughout in survivors (Supplement Fig. 2).

In line with the echocardiographic data, troponin T and BNP values were raised in all septic rats, markedly so in non-survivors from 6 h onwards (Fig. 3). IL-6 levels were significantly elevated, especially in non-survivors. However, by 72 h, no significant difference was observed between survivors and sham-operated controls (Supplement Fig. 2). IL-10 levels were consistently elevated in septic rats across all timepoints, with no distinction between survivors and non-survivors (Supplement Fig. 2).

### (ii) 24 h cardiomyocyte membrane cholesterol levels and adrenergic signaling in septic animals

Of the 48 septic rats in Study (b), a stroke volume cut-off value of < 0.19 ml at 6 h indicated 20 predicted survivors and 28 non-survivors at 72 h. At 24 h (experiment-end), one of the 20 (5%) predicted survivors and 13 of the 28 (46.4%) predicted non-survivors had died. All sham-operated control rats survived.

**Fig. 4:**
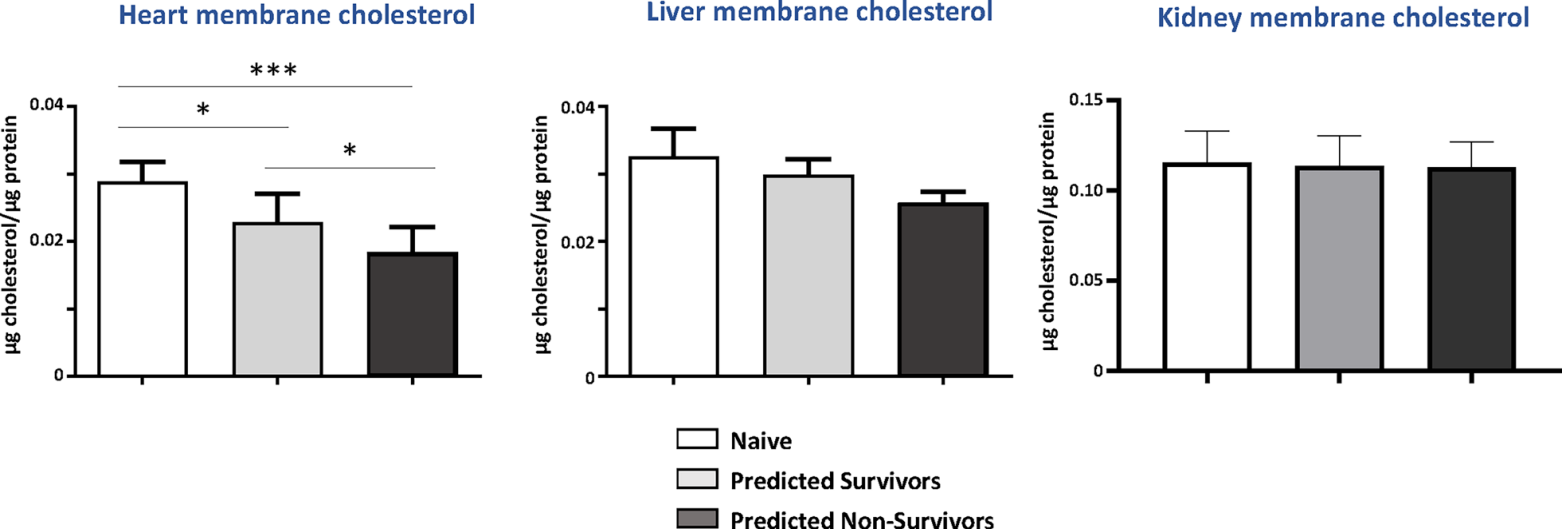
Membrane cholesterol levels in the heart, liver and kidney measured at 24 h. Membrane preps were prepared from the heart, liver and kidney tissue samples obtained in the in vivo experiment *Study (b) Tissue sampling at 24 h.* Cholesterol and protein concentration were measured by Amplex Red Cholesterol kit and Pierce™ BCA Protein Assay Kit correspondently. Data is shown as mean ± SD. One-way ANOVA with post-hoc testing (*p < 0.05; ***p < 0.001). Rat survival prediction based on 6-hour echocardiographic data. Group sizes ranged from 4–6 sham, 7–9 predicted survivors and 5–13 non-survivors

Tissue samples were thus available from those animals surviving to this timepoint, i.e. 6 sham-operated, 19 predicted survivors and 15 non-survivors. Cardiomyocyte membrane cholesterol levels at 24 h post-sepsis were significantly reduced (one-way ANOVA *p* < 0.0003) compared to sham-operated animals, more so in non-survivors (Fig. 4). The association between plasma cholesterol and cardiomyocyte membrane cholesterol (*r* = 0.43, *p* = 0.06) is shown in Supplementary Fig. 4. No significant difference was noted in liver or renal membrane cholesterol levels between septic and sham rats (Fig. 4). pERK activity was significantly higher in predicted survivors compared to non-survivors (Supplementary Fig. 5).

### (iii) impact of HDL- and liposomal cholesterol infusions on plasma and cardiomyocyte membrane cholesterol levels, and catecholamine responsiveness

**Fig. 5:**
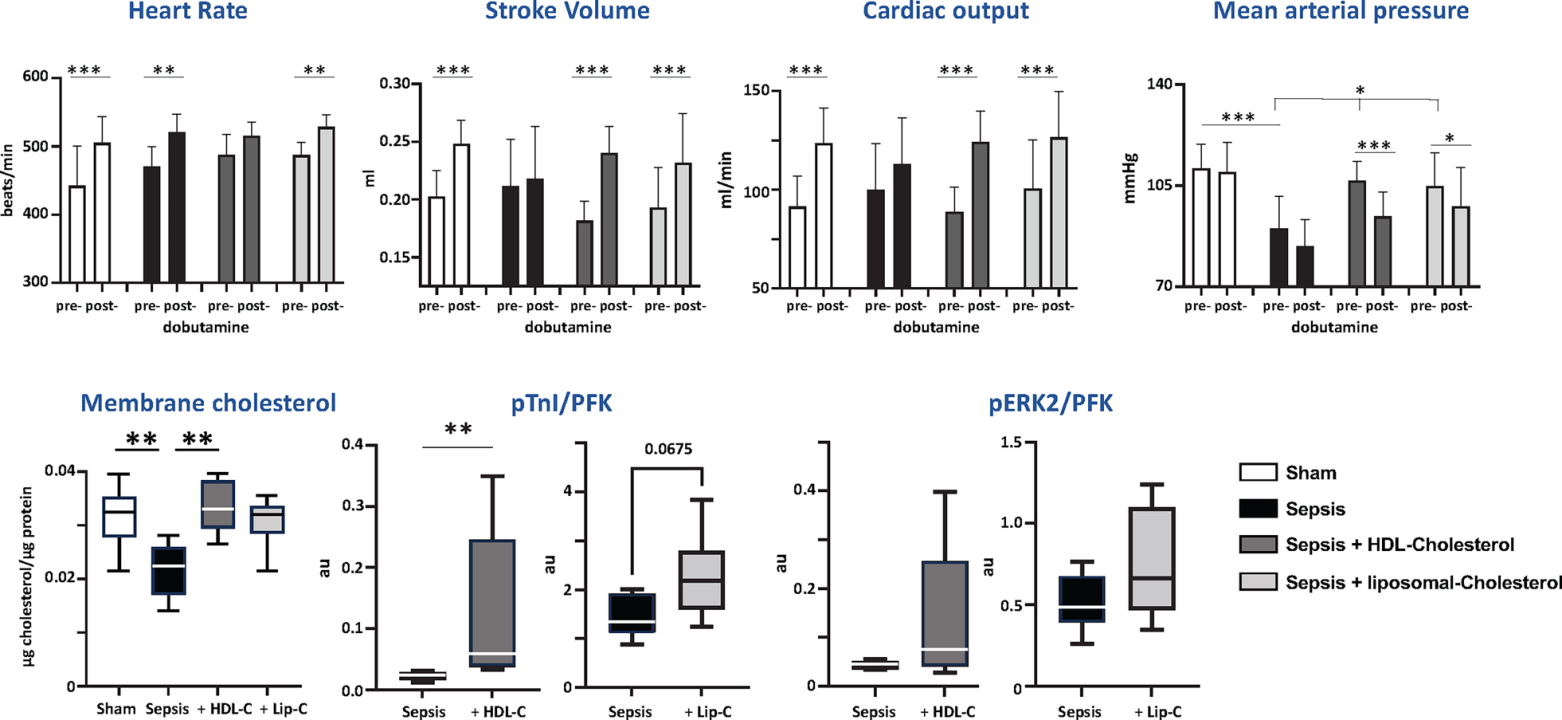
Impact of cholesterol treatment on catecholamine responsiveness, membrane cholesterol, and adrenergic signaling. Data shown are measurements made at 21 h in sham-operated, septic untreated, HDL-cholesterol and liposomal-cholesterol treated rats. *Hemodynamic data* shown as mean ± SD and analyzed by repeated measures 2-way ANOVA with post-hoc Tukey testing. Group sizes: sham (11, 12), placebo-treated sepsis (7), HDL-cholesterol-treated sepsis (6), liposomal-cholesterol-treated sepsis (8). *Membrane cholesterol data* shown as mean ± SD and analyzed by 1-way ANOVA and post-hoc testing (**p < 0.01). Group sizes: sham (4), untreated sepsis (5), HDL-cholesterol-treated sepsis (5), liposomal-cholesterol-treated sepsis (3). *Western blot* of pTnI and pERK2 ratioed to PFK analyzed by Wilcoxon Rank Sum test (*p < 0.05; **p < 0.01, ***p < 0.001); group sizes: 5–6

At 21 h, 11 of 14 (78.5%) placebo-treated septic animals, 11 of 12 (91.6%) septic animals treated with HDL-cholesterol, 9 of 15 (60%) liposomal cholesterol-treated animals, and all 13 (100%) sham animals had survived. After 15 h of cholesterol treatment, commencing at 6 h post-sepsis induction, no differences were seen in stroke volume or heart rate compared to untreated sepsis. However, blood pressure was significantly higher in both cholesterol-treated groups and restored to sham non-septic control levels (Fig. 5).

Dobutamine increased stroke volume and cardiac output to a significantly greater degree in cholesterol-treated septic rats compared to placebo-treated septic rats, with effects similar to those achieved in sham healthy controls (2-way analysis of ANOVA), (Fig. 5). The vasodilator effect of dobutamine was also more pronounced in cholesterol-treated compared to untreated septic animals. This enhanced beta-adrenergic activity of dobutamine with cholesterol treatment was associated with (i) restoration of cardiomyocyte membrane cholesterol to levels measured in sham-treated controls, and (ii) enhancement of the percentage change in phosphorylated troponin I and ERK2, markers of adrenergic sensitivity (albeit statistically significant only for pTnI after HDL treatment) (Fig. 5, Supplementary Fig. 6).

Sham animals responded to norepinephrine with a significant rise in blood pressure, but neither the untreated nor the cholesterol-treated groups showed any pressor effect (Supplementary Fig. 7). Plasma IL-6 and IL-10 levels were similar in cholesterol-treated groups compared to untreated septic animals (Supplementary Fig. 8). HDL-cholesterol and liposomal-cholesterol had contrasting effects on plasma and liver membrane cholesterol levels (Supplementary Fig. 9). Compared with untreated sepsis, plasma cholesterol tripled with HDL cholesterol treatment but was unchanged with liposomal cholesterol. By contrast, liver membrane cholesterol rose with liposomal cholesterol only, compared to control.

## Discussion

In this study, we have generated novel and potentially important mechanistic findings in our representative 72-hour fluid-resuscitated rat model of fecal peritonitis, namely, sepsis induced a decrease in cardiomyocyte membrane cholesterol, and the magnitude of which was associated with mortality risk. Restoring cardiomyocyte membrane cholesterol levels by infusion of exogenous cholesterol was associated with enhanced cardiovascular sensitivity to dobutamine and improved adrenergic signaling, but no impact was seen on the decreased pressor responsiveness to norepinephrine.

For obvious reasons, cardiomyocyte membrane cholesterol levels could not be measured in patients. Nonetheless, similarities were seen in plasma measurements between eventual survivors and non-survivors in both our 72-hour rat model and septic patients over the first four days of ICU admission, with a greater magnitude of change seen in non-survivors. These included falls in plasma cholesterol levels, elevated markers of cardiac injury (troponin) and ventricular dysfunction (BNP), altered hemodynamics (raised heart rate, decreased stroke volume) and elevated IL-6 and IL-10 cytokine levels. These findings underline the representativeness and likely relevance of this animal model, where mortality rates were comparable to our septic patients. Notably, in both species, hypocholesterolemia persisted for the duration of the study.

Hypocholesterolemia has been recognized in sepsis for more than a hundred years [42], with the magnitude of fall associated with an increasing mortality risk in multiple studies [9]. However, little attention has been paid to the clinical relevance of this finding. This is pertinent as cholesterol plays a crucial role in maintaining homeostasis, serving as the substrate for steroid and sex hormones, bile acids and Vitamin D, and offering anti-inflammatory, toxin-binding, and endothelium-protective properties [10, 13]. Cholesterol is also crucial in maintaining cell membrane integrity and functionality, regulating the activity of multiple transporters, ion channels and receptors. This includes G-coupled protein receptors [14–31], the focus of this current study examining myocardial depression in sepsis.

Low tissue/cell cholesterol levels are induced by inflammatory states, including neutrophils in patients with cystic fibrosis [43], skeletal muscle in a septic murine model [44] and skin in a murine pain model [45]. Importantly, our rat model showed that sepsis-induced changes in cholesterol levels varied by organ: cardiomyocyte membranes showed cholesterol loss, especially in predicted non-survivors whereas liver and renal membranes showed no significant differences. Whether hypocholesterolemia and decreased cardiomyocyte cholesterol are caused by decreased production, and/or increased metabolism or oxidation of cholesterol, or altered compartmentalization is subject to ongoing investigation.

A hallmark feature of sepsis is myocardial depression and decreased responsiveness to adrenergic stimulation [1–6]. This prognosticates for mortality in both septic patients and animal models [2–5, 32–34]. ß-adrenergic receptors are co-located with lipid rafts within the membrane where cholesterol is mainly concentrated [14–16, 22], and the cholesterol level determines the activity of both ß1 and ß2 receptors [25–27]. Experimental depletion of cardiomyocyte membrane cholesterol results in altered ß-adrenergic signaling [30, 31]. We have replicated this finding in H9C2 cardiomyocyte cell lines using methyl-beta-cyclodextrin and restored signaling with cholesterol repletion using cholesterol packaged within liposomes (unpublished data). The present study demonstrates improved cardiac performance with an increase in stroke volume and cardiac output in septic animals following dobutamine stimulation after cholesterol infusion. This effect was independent of the survival prediction category. The concomitant vasodilating effect also indicates enhanced ß_2_-adrenergic activity. We attempted to measure ß-adrenoceptor membrane density in lipid rafts, but several purchased antibodies did not yield reproducible results.

On the other hand, no impact was seen with either cholesterol preparation on the pressor response to norepinephrine in septic animals. The literature is inconsistent regarding the relationship between alpha-adrenergic signaling and membrane cholesterol content, showing enhanced sensitivity in both increased and decreased cholesterol states [46–48]. However, none of these studies were performed under septic conditions. We did not study changes in membrane cholesterol in vascular smooth muscle taken from arteriolar resistance vessels. However, the lack of response to norepinephrine does suggest that hyporesponsiveness to this pressor is either cholesterol-independent and/or other downstream mechanisms prevent restoration in response to cholesterol treatment.

Recent studies in septic animal models and patients have evaluated the potential therapeutic utility of either recombinant Apo-A1 or Apo-A1 mimetic peptides (the major component protein of HDL) [35–39], non-cholesterol-containing lipid emulsions [40] or liposomes containing sphingomyelin and cholesterol [41]. The thrust of these studies has been on the binding of bacterial toxins, with additional anti-inflammatory and endothelium-protective effects. In the current study, we targeted the delivery of cholesterol to cardiomyocytes with a focus on enhancing cardiac performance.

We compared a 15-hour infusion of an HDL- and a non-HDL-containing cholesterol preparation to determine whether the HDL component contributed to improvement. Both maintained blood pressure at the same level as healthy, sham-controlled animals and significantly higher than untreated septic controls. Dobutamine responsiveness and adrenergic signaling in the heart were also enhanced by both cholesterol treatments. Though not powered for survival, a reduced mortality signal was seen with HDL-cholesterol treatment compared to both untreated and liposomal-cholesterol animals. This may relate to the HDL component providing other, non-hemodynamic, effects, or, possibly, to harm related to the liposomes, particularly hepatic and immunological [49]. Of note, neither HDL- nor liposomal cholesterol preparations affected IL-6 and IL-10 levels compared to untreated septic animals. This implies that, at least in this model, neither HDL nor liposomal cholesterol had systemic immunomodulatory effects. Up to 90% of systemically injected liposomes are normally captured by the liver. A rise in liver membrane cholesterol was observed in our study with liposomal cholesterol treatment compared to control rats whereas a much higher plasma level was seen with HDL-cholesterol treatment. The rapid plasma clearance of liposomes through hepatic uptake could be reduced by pegylation or other strategies [49], which merits further investigation. Dose-finding studies also need to be performed to identify an optimal dosing regimen.

Even though LDL generally transports cholesterol from liver to peripheral organs, we used HDL in the current study. Cardiomyocytes have a limited uptake of LDL-cholesterol [50], whereas heart tissue expresses the SR-B1 (the Scavenger Receptor Class B type I (SR-B1) receptor for high-density lipoproteins (HDLs) facilitating uptake of its esterified cholesterol content [51, 52]. The increase in cardiomyocyte membrane cholesterol with HDL-cholesterol treatment provides confirmatory evidence of myocardial uptake.

Other than enhancing adrenergic signaling, other potential mechanisms by which cholesterol treatment could enhance inotropic responsiveness, include (i) improved effects on ß-dystroglycans as cholesterol depletion in rat skeletal muscle fibers uncouple these glycoproteins from lipid rafts and decrease contractility [53], (ii) reversal of a fall in basal shortening and [Ca^2+^] transients, as was identified in isolated primary cardiomyocytes depleted of cholesterol [54], (iii) activation of other G-protein coupled receptors, e.g. for oxytocin, endothelin-1, serotonin-1 A and vasopressin, that are also dependent on membrane cholesterol levels [55, 56], or (iv) enabling membrane repair [57, 58].

Some limitations should be highlighted. Antibiotics were not given in the rat model nor source control performed, as would be done in human sepsis. In a long-term (100-hour) murine cecal ligation and puncture model given fluids but lacking source control, Lewis et al. demonstrated a prolonged survival time with earlier antibiotics yet all animals still died [59]. In our model, postmortem examination of the 44% of animals surviving to study end (72 h) - and clinically much improved - showed spontaneous walling-off by the peritoneum of the fecal slurry and much less inflammation compared to those not surviving. Only male rats were used in this study. Addition of a female subgroup to draw sex-based comparisons would considerably increase numbers of animals needed, going against the 3 Rs (Replacement, Reduction, and Refinement) core principles of animal research. Subsequent planned studies will however comprise targeted experiments using both sexes.

## Conclusions

In this study, we demonstrated similar severity-related changes in both septic patients and rats with altered hemodynamics and elevated markers of ventricular dysfunction associated with decreases in plasma cholesterol. Further studies in this clinically relevant rat septic model showed differential effects of sepsis on tissue cholesterol levels, with significant decreases in heart tissue, lesser change in the liver, and no change in the kidney. The reduction in cardiomyocyte membrane cholesterol also prognosticated for poor outcomes, and was associated with impaired cardiac functionality, decreased adrenergic signaling and reduced responsiveness to dobutamine. Restoring myocardial cholesterol levels by treatment with different cholesterol formulations was able to rescue myocardial function. Further studies are warranted to explore underlying mechanisms and the potential of cholesterol treatment.

## Supplementary Information

Supplementary Material 1.

## Data Availability

Data generated in this study are included in the main paper or supplementary material. Further information can be provided by the corresponding author on reasonable request.
