# Supplementary material for: Sepsis-induced hypocholesterolemia is linked to low cardiomyocyte membrane cholesterol and impaired catecholamine responsiveness
Source: Crit Care. 2025 Sep 25;29:399. doi: 10.1186/s13054-025-05638-7 (PMC12465896; doi:10.1186/s13054-025-05638-7)

**Supplementary materials**

**Index:**

**1. Supplementary Figures 1-9**

**2. Patient study**

**Design**

**Supplementary Table 1: Patient demographic and clinical data**

**Human patient blood measurements**:

**3. Rat fecal peritonitis study**

**Design**

**Supplementary Table 2: Clinical severity scoring system**

**Ex vivo tests on rat blood and tissue samples**

**Supplementary Table 3: Antibodies**

**4. Preparation of liposomal cholesterol**

**Supplementary Figure 1: 72-hour rat fecal peritonitis study – Kaplan-Meier survival curve**

**
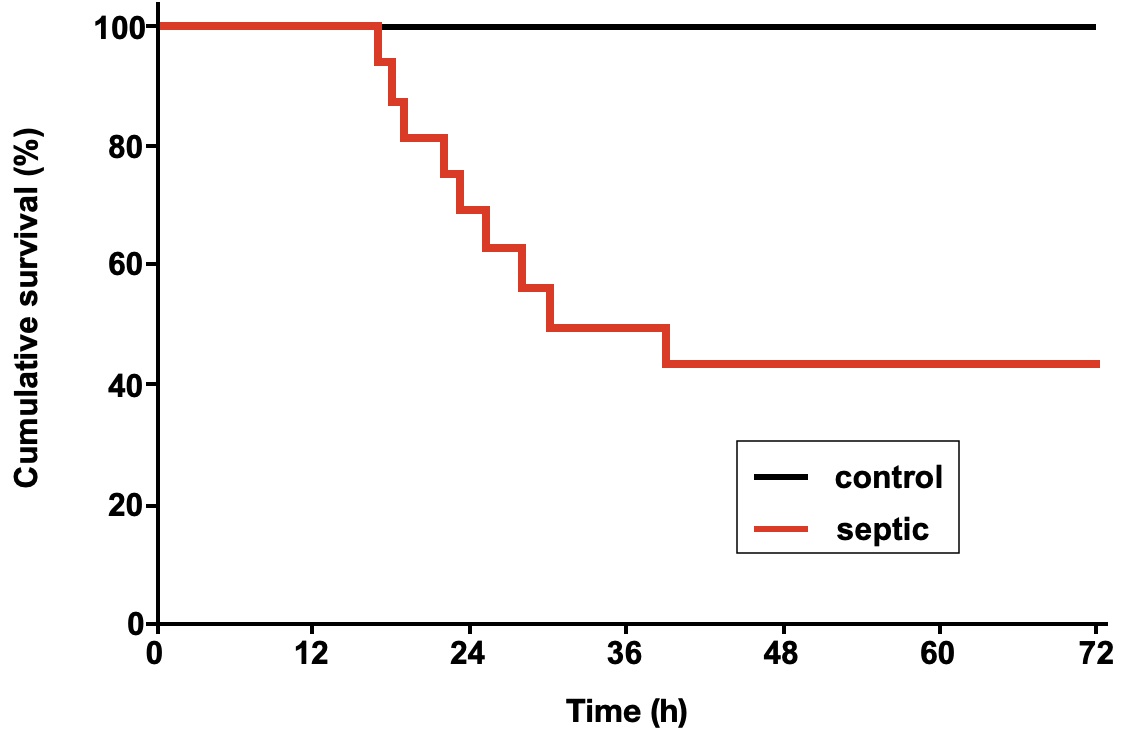
**

**Supplementary Figure 2: Plasma triglyceride, IL-6 and IL-10 levels in septic patients and rats**

**
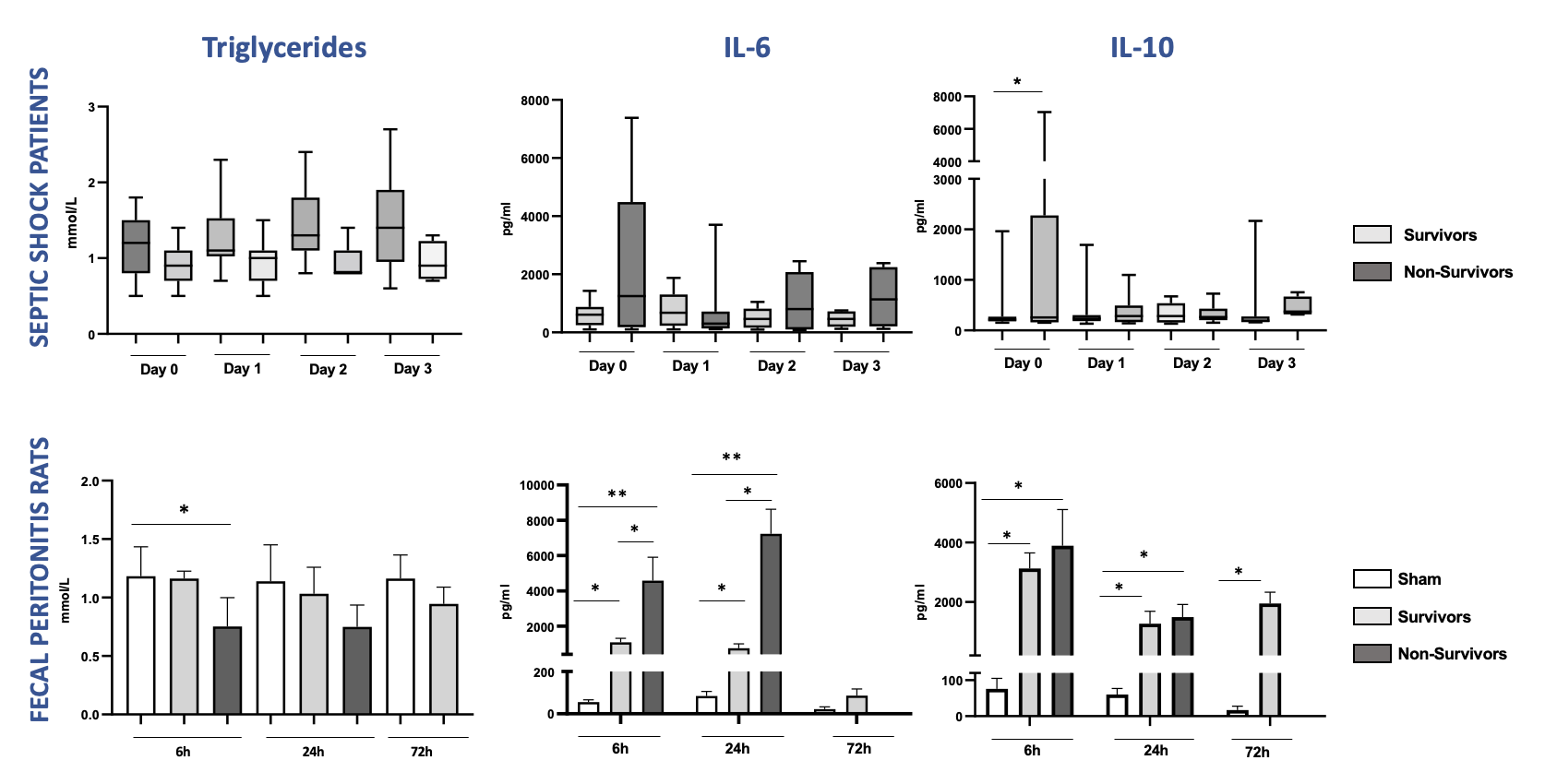
**

Data shown as mean ± SD (parametric) or box and whisper plots (non-parametric). Repeated measures 2-way ANOVA with post-hoc testing shown if overall ANOVA positive (*p<0.05; **p<0.01). Patient group sizes: 13, 12, 9 and 8 survivors on Days 0-4 respectively; 11, 9, 6, 4 non-survivors on Days 0-4 respectively. Rat group sizes varied from 5-8 (six sham, six survivor, eight non-survivor at 6h and five at 24h and 72 h).

**Supplementary Figure 3: Associations between cholesterol (total and HDL), BNP and troponin T over Days 0-3 of ICU admission**

**
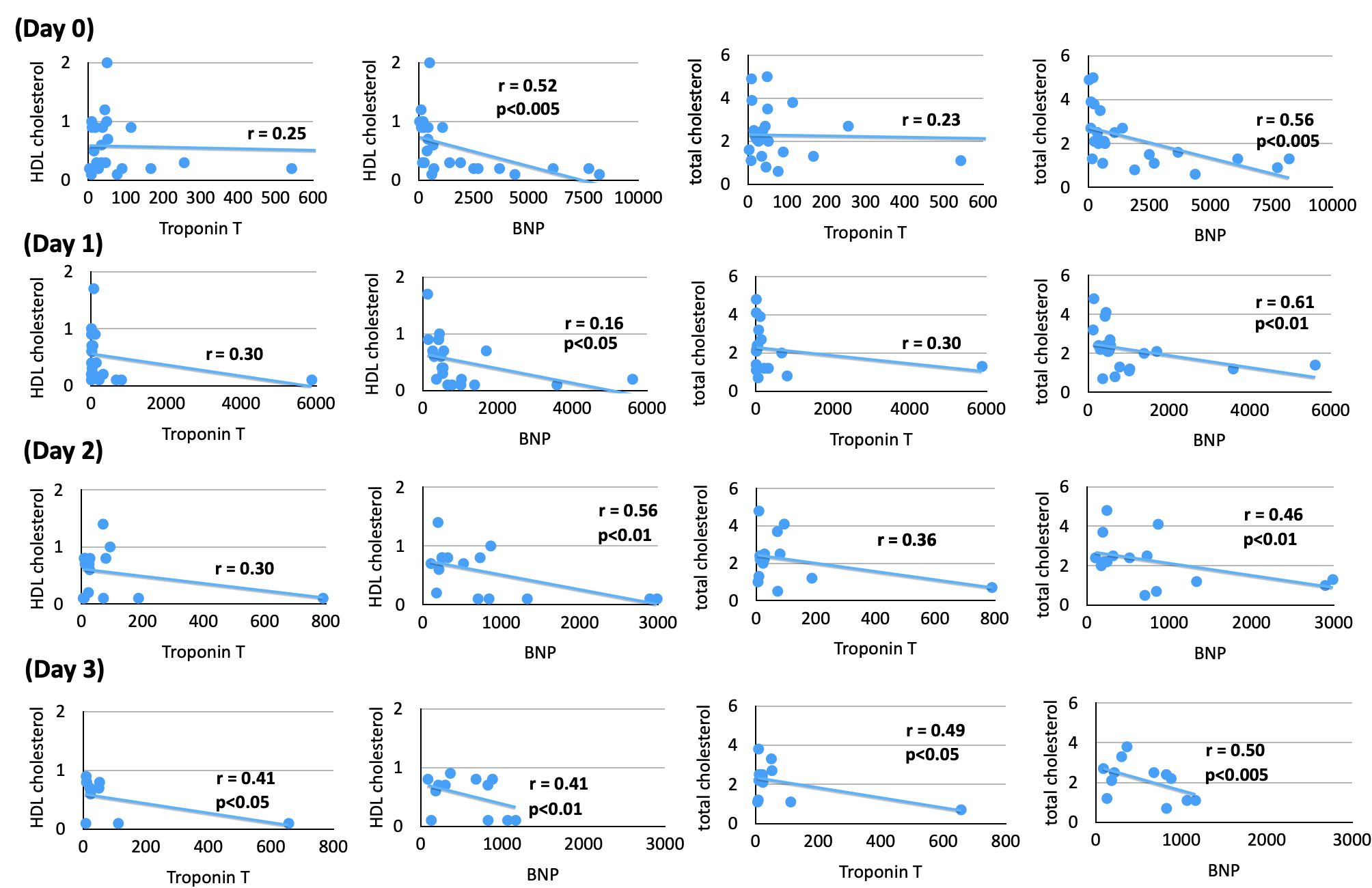
**

**Supplementary Figure 4: Relationship between plasma cholesterol and cardiomyocyte membrane cholesterol at 24 hours**

**
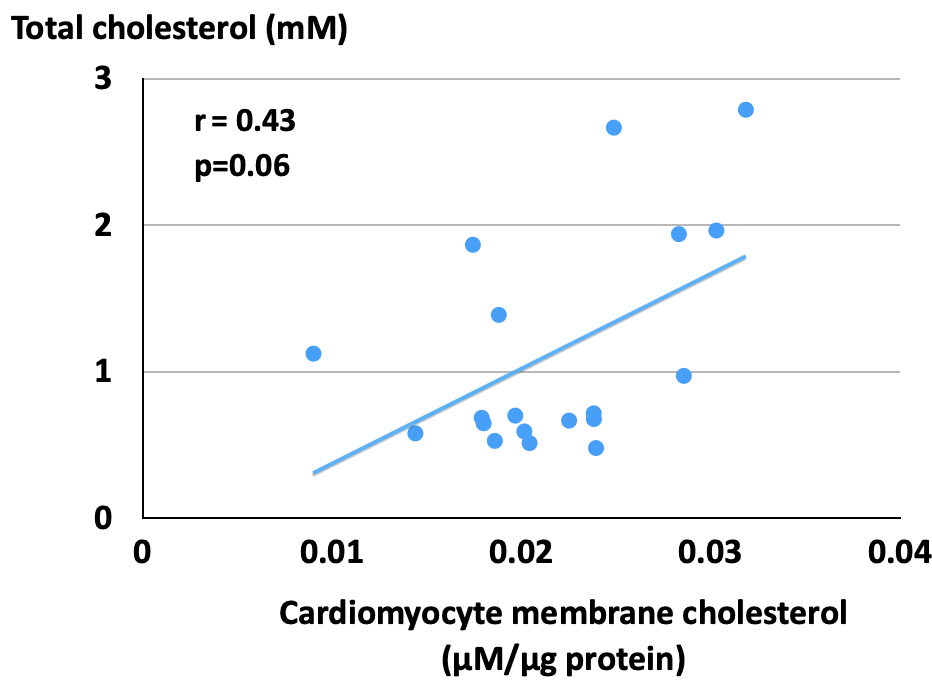
**

**Supplementary Figure 5: Adrenergic signaling in cardiac tissue sampled after 24 hours of sepsis.**

**
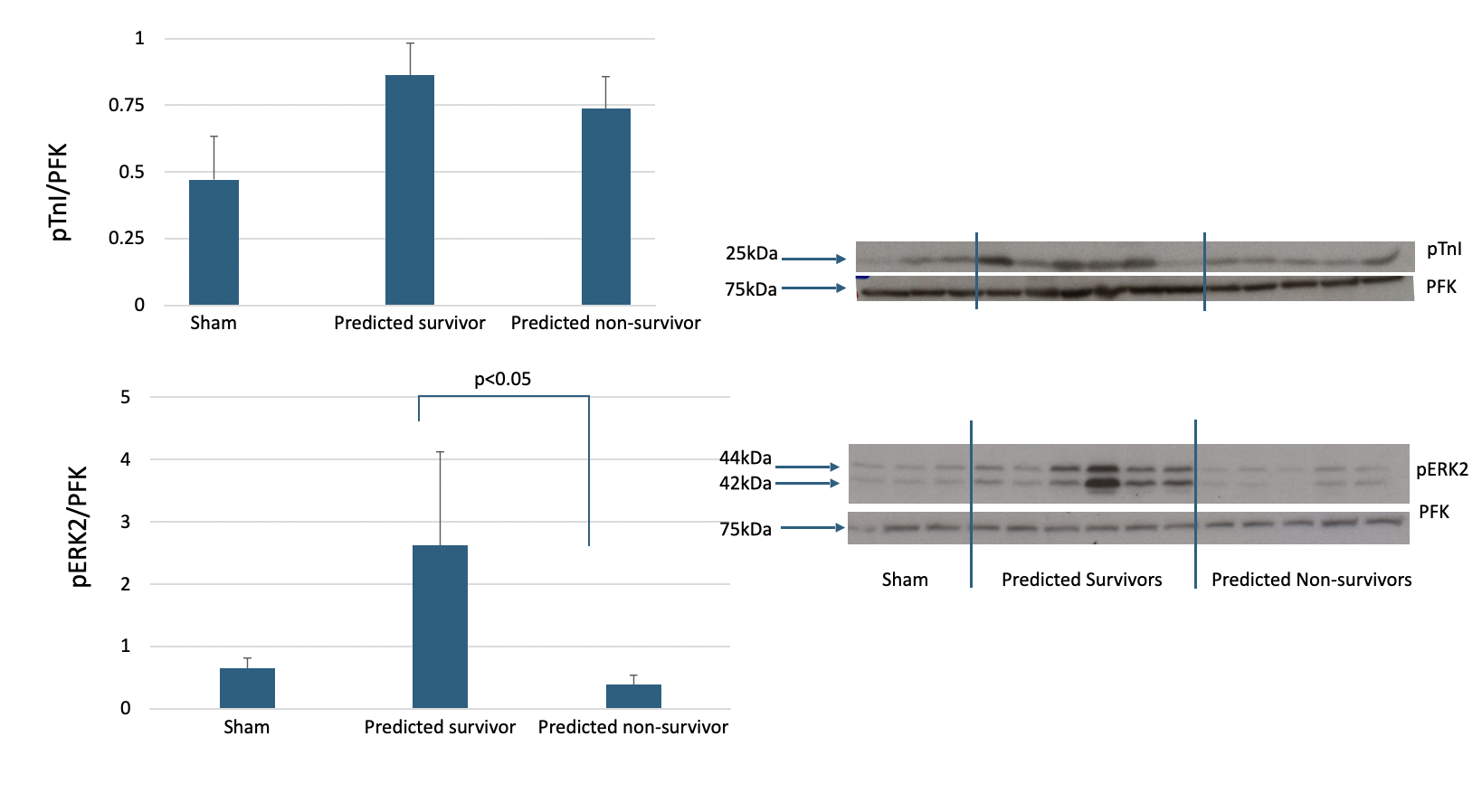
**

phosphoTroponin I and phosphoERK2 protein levels in predicted survivor and non-survivor septic rats (identified at 6 hours by echocardiography), and sham control rats. Specimen Western blots shown in right panel

**Supplementary Figure 6: Specimen Western blots for pTroponin I and pERK2 in untreated sepsis and following HDL-cholesterol or liposomal cholesterol infusion**

**
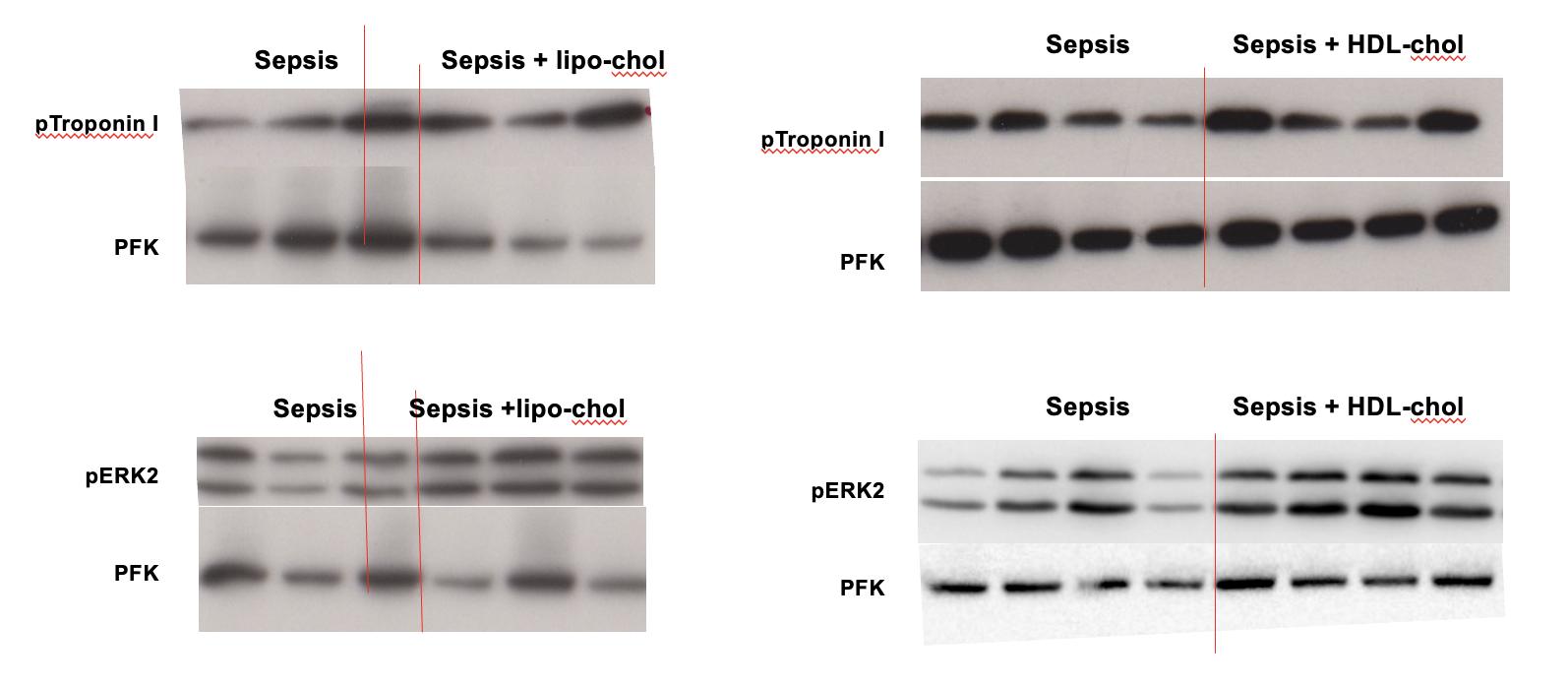
**

**Supplementary Figure 7: Hemodynamic response to norepinephrine in sham, septic rats, and septic rats treated with either HDL- or liposomal-cholesterol**

**
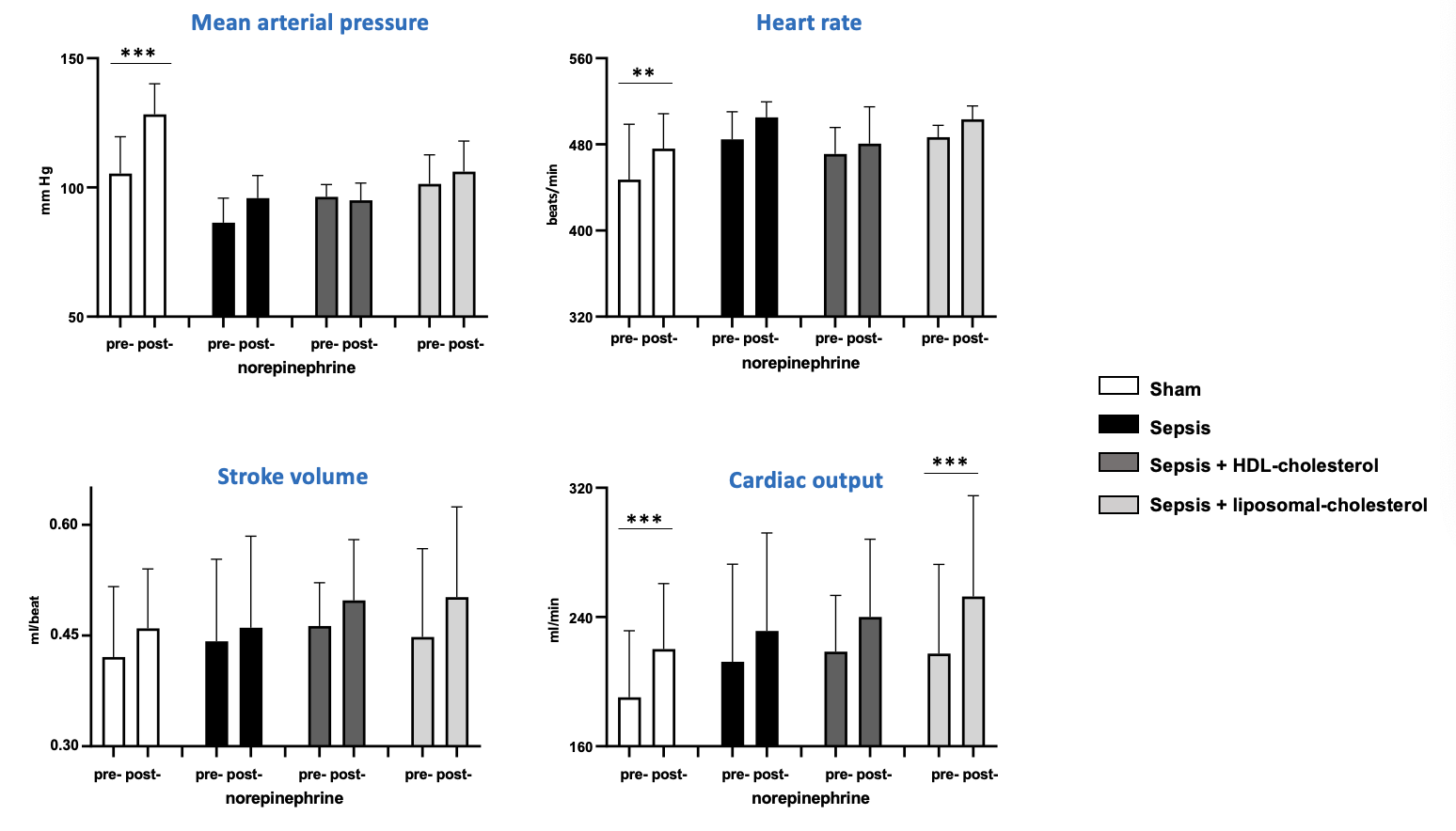
**

Hemodynamic data shown as mean ± SD and analysed by repeated measures 2-way ANOVA with post-hoc Tukey testing where positive. Group sizes: sham 11-12 , untreated sepsis 7, HDL-cholesterol-treated sepsis 6, liposomal-cholesterol-treated sepsis 8. *p<0.05; **p<0.01, ***p<0.001

**Supplementary Figure 8: IL-6 and IL-10 levels in sham, septic rats, and septic rats treated with either HDL- or liposomal-cholesterol**

**
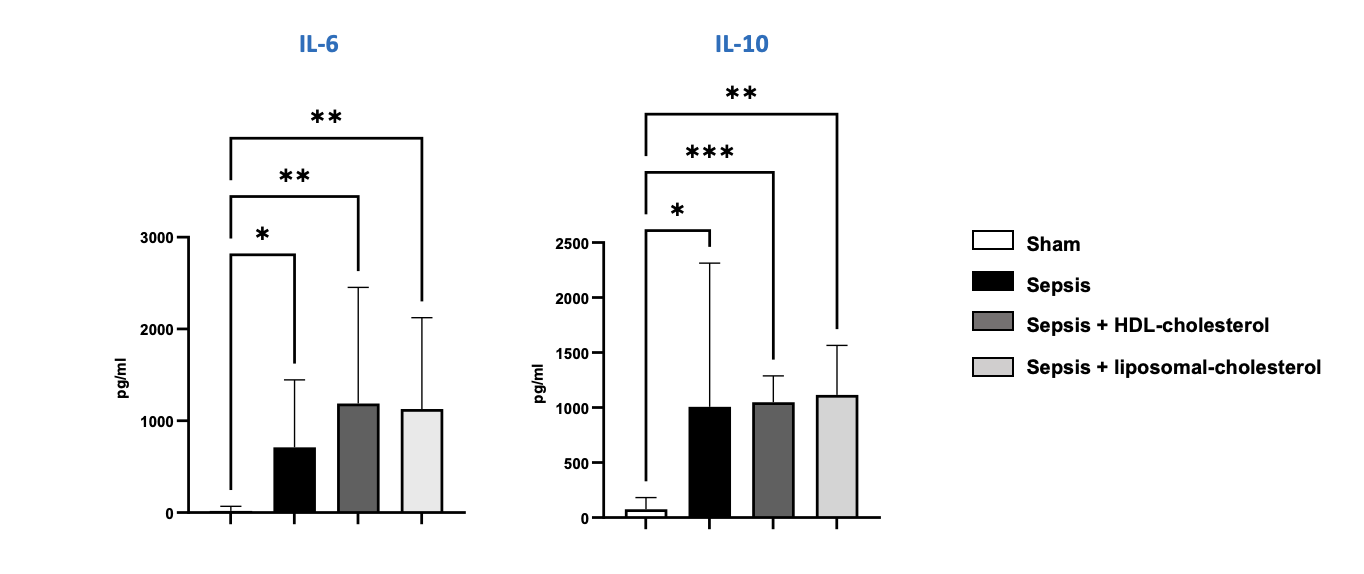
**

Data shown are measurements made at 21 hours in sham-operated, septic, placebo-treated, HDL-cholesterol and liposomal cholesterol-treated rats (with cholesterol infusion commenced at 6 hours). Data are presented as mean ± SD and analysed by 1-way ANOVA (p<0.01 for IL-6 and p<0.001 for IL-10) and post-hoc testing. Group sizes: sham 9, untreated sepsis 7, HDL-cholesterol-treated sepsis 6, liposomal-cholesterol-treated sepsis 8. *p<0.05; **p<0.01, ***p<0.001

**Supplementary Figure 9: Plasma and liver membrane cholesterol in sham, septic rats, and septic rats treated with either HDL- or liposomal-cholesterol**

**
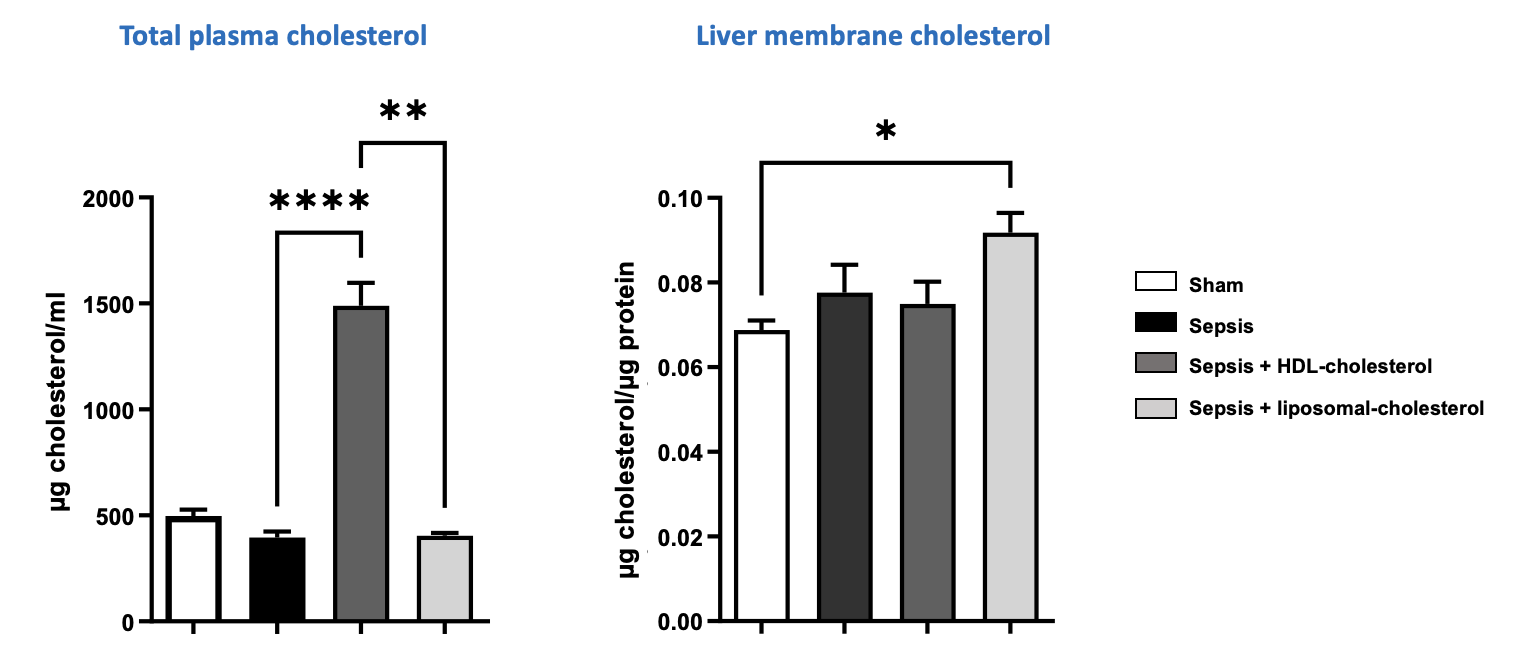
**

Data shown are measurements made at 21 hours in sham-operated, septic placebo-treated, HDL-cholesterol and liposomal cholesterol-treated rats (with cholesterol infusion commenced at 6 hours). Data are presented as mean ± SD and analysed by 1-way ANOVA (p<0.001 for plasma cholesterol and p<0.05 for liver cholesterol) and post-hoc testing. Group sizes: plasma: sham 9, placebo-treated sepsis 7, HDL-cholesterol-treated sepsis 6, liposomal-cholesterol-treated sepsis 8. Liver: sham 5, placebo-treated sepsis 4, HDL-cholesterol-treated sepsis 5, liposomal-cholesterol-treated sepsis 4.*p<0.05; **p<0.01, ***p<0.001

**3. Patient study**

Fifty-one patients were enrolled into the STRESS study, a prospective, observational cohort study of critically ill adult patients admitted to the ICU at University College London Hospital (UCLH). Of these, 13 patients were diagnosed with intra-abdominal sepsis and 14 patients with community-acquired pneumonia. No interventions other than the standard of care were administered. Demographic, clinical and laboratory data were recorded daily; blood sampling was undertaken from arterial lines (or, if no arterial line was available, from a central venous catheter or venepuncture) on the day of admission (Day 0) and, subsequently, on Days 1, 2, and 3. Other than admission blood samples, all remaining samples were collected between 08.00 and 09.00 each morning. Routine laboratory tests were recorded from the patient’s record sheet. Additional 20 ml blood samples were taken for this study and placed into appropriate EDTA (BD Vacutainer Lavender), clot activator and gel (BD Vacutainer Gold) tubes (all BD Diagnostics, Franklin Lakes, NJ), plain and EDTA-containing Eppendorf tubes. Samples were stored on ice immediately after collection and subsequently centrifuged at 6500 g for 10 min. Plasma or serum supernatants were decanted into 200 µl aliquots, snap-frozen into liquid nitrogen and stored at -80°C prior to batch analyses.

**Supplementary Table 1: Patient demographic and clinical data**

|  | Survivors (n=15) | Non-survivors (n=12) |
| --- | --- | --- |
| Age (y) | 62.6±12.5 | 63.5±10.9 |
| Male sex (%) | 9 (60%) | 8 (67%) |
| Weight (kg) | 78 ± 11 | 74 ±11 |
| 1^st^ 24 hour APACHE II score | 14.9 ± 4.8 | 19.2 ± 6.7 |
| SOFA score | 7.3 ± 2.2 | 9.6 ± 3.3 |
| Intra-abdominal sepsis | 8 | 5 |
| Community-acquired pneumonia | 7 | 7 |
| Admission source   - Emergency department - Ward - Operating theatre | 7  6  2 | 6  2  4 |
| Day 0 lactate (mmol/l) | 1.9 ± 2.1 | 5.9 ± 4.8 |
| Vasopressor agent (any)  Day 0  Day 1  Day 2  Day 3 | 12 (80%)  8 (53%)  7 (47%)  3 (20%) | 12 (100%)  11 (100%) (n=11)  5 (60%) (n=10)  4 (50%) (n=8) |

**Human patient blood measurements**: Total HDL and LDL/VLDL cholesterol, triglyceride (AU5800 analyser, Beckman Coulter, High Wycombe, UK) and high-sensitivity troponin-T levels (high-sensitivity cardiac troponin T electroluminescence immunoassay (Roche Diagnostics, Basel, Switzerland) were measured by the Clinical Chemistry Department, Royal Free Hospital, London, UK. The lab provided normal ranges for these assays. B-type natriuretic peptide (BNP) (E-EL-H0598, Elabscience) and the cytokines IL-6 and IL-10 (DY-506 and DY522, R&D Systems) were measured in our lab by ELISA following the manufacturers’ instructions. For all 96-well microtiter plate readings, signal quantification was performed with a Synergy 2 plate reader (Biotek, Winooski, VE, USA) set at the appropriate wavelength. Gen5 analysis software (Biotek) was used to produce a best-fit curve from the optical density values of the ELISA reference standards. Standard curves with an R^2^ approaching 1 were repeatedly obtained. All data files were exported to Prism (GraphPad, La Jolla, CA, USA) for analysis.

**4. Animal fecal peritonitis**

**Model set-up**

Animal experiments were conducted using a long-term fluid-resuscitated rat model of sepsis from fecal peritonitis. Studies were undertaken following local UCL ethics committee approval under the Animals (Scientific Procedures) Act 1986. The UK Home Office granted personal and project licenses for the animal work. Experimental designs for each study is shown in Supplementary Fig 1.

Male Wistar rats (Charles River, Margate, Kent) weighing between 325-400g were housed in cages of four for a week prior to experimentation. They had unimpeded access to food and water and were kept in a room with automated lighting to simulate a normal day-night cycle.

Rats underwent instrumentation and echocardiography under brief general anesthesia using isoflurane (Baxter Healthcare, Thetford, Norfolk, UK). An anesthetic vaporizer (Vet-Tech Solutions, Congleton, Cheshire, UK) and air pump (TetraTec APS400, Tetra GmbH, Melle, Germany) were used to administer the anesthetic. Rats were placed in a plastic induction chamber and anesthesia induced using 5% isoflurane. Maintenance anesthesia was achieved using 2% isoflurane with animals spontaneously breathing through a nose cone. Rats were then positioned supine on a heated mat with continuous monitoring of core temperature via a rectal thermometer (TES 1319, TES Electrical Electronic Corp, Taipei, Taiwan). Body temperature was maintained between 36.0-37.5°C while under anesthesia.

A chemical depilatory cream was used to remove hair from the neck and chest (Nair, Church and Dwight, Folkestone, Kent). Preoperative skin preparation was completed using a commercially available mix of 2% chlorhexidine gluconate and 70% isopropyl alcohol (ChloraPrep, CareFusion, San Diego, CA). A sterile skin drape was used, and all surgical procedures were performed with strict aseptic technique.

A 2 cm vertical incision in the center of the neck was used to obtain access to the right internal jugular vein and the left common carotid artery. Both vessels were cannulated with 0.96 mm outer diameter PVC tubing (Biocorp Ltd, Huntingdale, NSW, Australia) and secured in place with two 3-0 silk sutures. This allowed continuous blood pressure monitoring and blood sampling through the arterial line, and fluid resuscitation through the venous line. Both lines were tunneled subcutaneously to the nape of the neck and attached to a dual-channel swivel and button tether system (InsTech Solomon, Plymouth Meeting, PA, USA) which was secured to the skin using four 2-0 silk sutures. The skin incision site was sutured using 3-0 silk sutures. To provide analgesia rats were given a subcutaneous injection of 0.05 mg/kg buprenorphine (Vetergesic, Reckitt Benckiser, York, UK) prior to recovery.

Following recovery from anesthesia, animals were individually housed in metabolic cages with the swivel-tether system attached to a balancing arm. This allowed the rats unfettered movement in their cages with access to food and water *ad libitum*. Room temperature was kept at 21.1 ± 1.1°C, humidity between 40-70%, and a 12-hour light-dark cycle was maintained.

Both venous and arterial lines were flushed continuously to maintain patency with 0.1 ml/h of 0.9% saline (Baxter Healthcare, Thetford, Norfolk, UK). The arterial line was connected to a pressure transducer (SensoNOR SP844, Memscap, Skoppum, Norway) and blood pressure recorded continuously onto a Powerlab system (AD Instruments, Chalgrove, Oxon, UK).

Stool samples were obtained from six healthy non-vegetarian human volunteers and pooled together. Samples were collected in styrofoam containers, weighed and stored on ice. Collected fecal material was continuously fumigated with nitrogen in order to maintain an anaerobic environment. The collected material was diluted 1:1 with a suspension of thioglycolate (14.5g / 500mL distilled water) and catalase (0.19 mg / 100mL) to optimize bacterial growth and inactivate reactive oxygen species. For cryoconservation 10% glycerine was added and the suspension homogenized under anerobic conditions. The resulting material was divided into 0.5 ml aliquots and frozen at -80°C. Prior to use, each aliquot was thawed and diluted 1:7 with 0.9% saline to form fecal slurry.

Prior to recovery from anesthesia, sepsis was induced in the rats by intraperitoneal (i.p.) injection of 4-7 µl/g body weight fecal slurry using a 19-gauge needle injected in the right lower quadrant of the abdomen (later experiments utilized a new batch of slurry and required a higher dose to achieve the same severity – an approximate predicted 30-40% mortality at 72 hours. Sham operated control animals received no i.p. placebo injection to avoid inadvertent bowel perforation.

Fluid resuscitation (10 ml/kg/h) was initiated 2 hours’ post-injection of faecal slurry. A 50:50 mix of Hartmann’s solution and 5% glucose were infused via the indwelling venous catheter. This fluid infusion rate was reduced to 5 ml/kg/h at 48 hours. An identical fluid regimen was used for control animals. Glucose was added to avoid hypoglycemia as unwell animals did not eat.

Illness severity was assessed using a clinical scoring system we developed in conjunction with the UCL Biological Services Unit, and subsequently validated. All rats were assessed at least four times daily throughout the course of the experiment. Increasing clinical severity resulted in more intensive monitoring. Animals scoring ‘3’ were deemed ‘critical’ resulting in termination if on a moderate protocol and scoring ‘4’ were promptly culled (for survival analysis).

**Supplementary Table 2: Clinical severity scoring system**


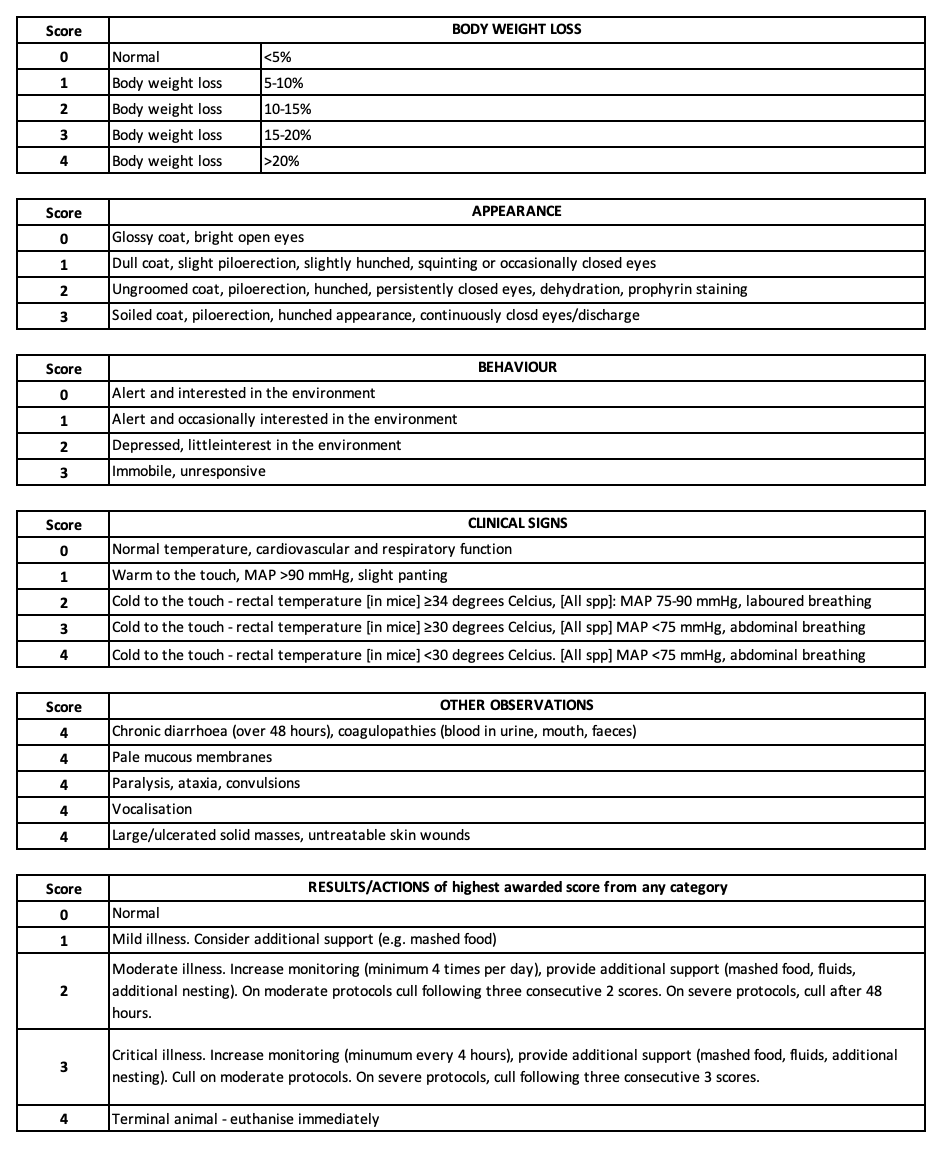


Transthoracic echocardiography (TTE) was performed at baseline prior to insertion of vascular catheters. TTE was also performed at 6 hours after induction of sepsis, at 24, 48 and 72 hours. Previous work in the host lab has shown echocardiography-derived stroke volume to be an accurate predictor of 72-hour mortality as early as 6 hours after the septic insult; at this timepoint surviving and non-surviving animals cannot be clinically distinguished. Those animals that succumb usually do so between 18-36 hours; survivors show clear signs of recovery (appearance, activity, feeding) at the 72-hour study endpoint.

TTE was performed using a Vivid 7 Dimension device (GE Healthcare, Bedford, Beds) and a 10MHz sector transducer (Vivid 10S, GE Healthcare). All variables were recorded under a brief period of 1.2% isoflurane anesthesia. A parasternal long-axis view of the left ventricle was obtained. The diameter from the aortic annulus to the apex was measured during end-systole and end-diastole.

Aortic blood flow was measured using pulsed wave Doppler at the point of bifurcation of the right carotid artery from the aortic arch. Blood flow direction was confirmed using colour Doppler. The area under each waveform obtained by pulsed wave Doppler is the velocity-time integral (VTI). Due to variability of heart rate with respiration, an average VTI was taken after measuring the area under 6 consecutive waveforms. Heart rate (HR) was calculated by measuring the time between the start of each Doppler waveform over 6 consecutive cycles. HR ranged from 350-500 beats/min during these experimental conditions. Stroke volume (SV) was calculated as 0.25 π *d*^2^ (*VTI*) where *d* is the aortic diameter. Aortic diameter was previously measured in rats of the same age and weight as those used in these experiments and found to be very similar between them with a mean diameter of 0.26 cm. Cardiac output (CO) was calculated as *SV* x *HR*

*Catecholamine responsiveness tests.*

After a 30-minute stabilisation period animals had baseline measures made of blood pressure, and (by echocardiography) stroke volume and heart rate. Dobutamine 10 µg/kg/min was then infused for 10 min followed by repeat echocardiography and BP recording. After a 30-min washout period this procedure was repeated but with a 10-minute infusion of norepinephrine 0.5 µg/kg/min.

Blood samples were taken at appropriate timepoints (Supplement Fig 1) according to the study design. At experiment end animals were terminally anesthetised and then euthanised by exsanguination with heart, liver and kidney tissue samples rapidly taken and snap-frozen in liquid nitrogen. Blood was centrifuged and separated to collect aliquots of plasma and serum that were also kept frozen for subsequent analyses.

**4. Ex vivo tests on rat blood and tissue samples**

***4.1. Blood samples***

Total, HDL- and LDL-cholesterol and triglycerides in the 72h study (Study 1) were measured at the Royal Free Hospital (as above). In other studies, total and non-esterified plasma cholesterol were measured by Amplex Red assay (Invitrogen A12216).

Troponin T and BNP measurement: human Troponin T was measured in the Royal Free Hospital (London, UK) biochemistry laboratory using a 4^th^ generation high-sensitivity cardiac troponin T electrochemiluminescence immunoassay (ELISA) (Roche Diagnostics, Basel, Switzerland). Rat troponin T measurement was performed using a sandwich ELISA (E-EL-R0151, Elabscience Biotechnology Co, Beijing, China). BNP levels were measured by competitive ELISA in the rats (RAB0386, Sigma-Aldrich) and human samples (EELH0598, Elabscience) according to the manufacturer’s protocol.

**Cholesterol measurement in the tissue membranes**: Using mortar and pestle frozen tissue was pulverized in liquid nitrogen; appr 50mg (1 spoon of tissue powder) were homogenized in 1 ml ice-cold **Membrane buffer** (10mM Tris HCl pH 7.4 50mM NaCl, protease inhibitors) in 7ml Dounce homogenizer with tight pestle, 20 strokes. Homogenates were transferred into Eppendorf tubes, centrifuged 2 times at 4C 10min 1000g. Both times pellet was discarded, supernatant transferred into new tubes. 500µl supernatant was transferred into a centrifuge tube (Backman Counter tube Ultra Clear 9/16 x 3 1/2in (14x89mm)), 50mM Tris HCl pH 7.4 was added till the rims. Samples were centrifuged on a Beckman ultracentrifuge for 1.5 hours at 4 °c, 30000 rpm, Rotor SW41. After centrifugation, supernatant was discarded, pellet was dissolved in 300μl **Sample buffer** (10mM TrisHCl pH 7.4, 50mM NaCl, 1mM EDTA, 1mM EGTA, 0.1% Triton x100) vortexed vigorously and passed 20 times through the 27th G needle insulin syringe, centrifuged at 4C 10min 14000rpm. Protein concentration in membrane prep was determined by BCA assay (A55864, ThermoFisher Scientific), cholesterol - by Amplex Red Cholesterol Assay kit

**Protein lysates for WB**: Frozen heart or liver tissues were crashed with hammer on dry ice, a piece of tissue 30-50mg was transferred into 2ml Precellys O-ring tube containing ice-cold 1ml RIPA buffer with protease and phosphatase inhibitors (Roche Complete Ultra 05892970001, Phosphostop 04906845001 1 tablet/10ml buffer). Tissue was homogenized using MiniLys homogenizer 5x20sec maximum speed cycles, after each cycle a tube was chilled on ice. After homogenization lysates were incubated 30min on ice, next centrifuged on bench centrifuge 10min at 4C and 14000rpm. The supernatants were transferred into new tubes; a protein concentration of the lysates was measured by BCA assay (Thermo Fisher Scientific). Lysates were stored at -80C till further analysis. The volume of protein lysate containing 40μg proteins was adjusted to 7μl with RIPA buffer and mixed with 7μl 2xfold loading buffer (100mM TrisHCl pH 6.8, 4% SDS, 20% Glycerol, 0.2% Bromphenol blue, 200mM β-mercaptoethanol fresh added). Before loading on the gel WB samples were boiled at 95C 5min.

**Western Blot analysis:** Samples were loaded onto 10% SDS-PAGE, proteins separated in an electric field, transferred to Immobilon-P (Millipore, IPVH00010), and blocked for 1 hour at RT with 5% BSA (Sigma A2153) in TBST buffer + 0.02% NaN_3_. The membrane was cut into longitudinal strips for incubation with various primary antibodies (see Suppl Table 3) at 4°C overnight in 5% BSA in TBST+ 0.02 NaN_3_ with gentle agitation. Secondary antibodies were diluted in 5% BSA in TBST and added for 1 hour at room temperature with gentle agitation. Amersham HyperFilm ECL (GE Healthcare 28906837) and Clarity Western ECL (Bio-Rad CatN#170-5061) were used for development. Results were evaluated by Image Studio Lite version 5.2.

**Supplementary Table 3: Antibodies**

| Name | Predicted Size (kDa) | Dilution | Host/Secondary Abs |
| --- | --- | --- | --- |
| Phosphotroponin I Cardiac Ser 23/24  Cell Signalling #4004 | 28 | 1:1000 in BSA | Rabbit / Daco anti-rabbit  P0448 1:10.000 |
| PFK  ProteinTech  55028-I-AP | 75 | 1:10000 in BSA | Rabbit polyclonal/ Daco anti-rabbit  P0448 1:10.000 |
| pERK1/2  Cell Signalling #9101 | 42/44 | 1:1000 in BSA | Rabbit / Daco anti-rabbit  P0448 1:10.000 |

**Liposome preparation and characterisation**


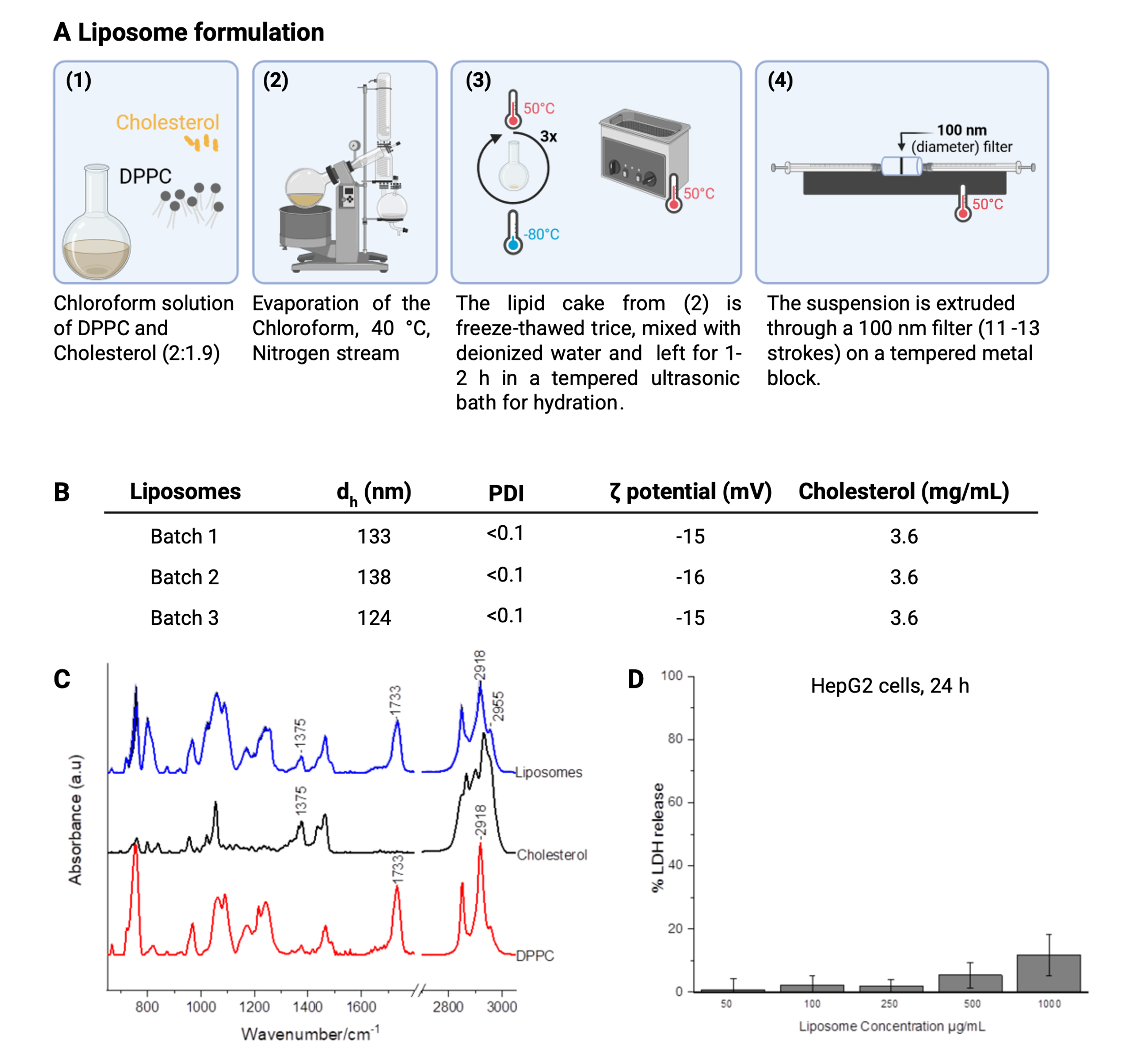

Supplement: Supplementary file 1 — Supplementary Material 1. [file 13054_2025_5638_MOESM1_ESM.docx]
